# Classification of Breast Cancer Nottingham Prognostic Index Using High-Dimensional Embedding and Residual Neural Network

**DOI:** 10.3390/cancers14040934

**Published:** 2022-02-13

**Authors:** Li Zhou, Maria Rueda, Abedalrhman Alkhateeb

**Affiliations:** 1 School of Computer Science, University of Windsor, Windsor, ON N9B 3P4, Canada; zhou18j@uwindsor.ca; 2 Department of Chemistry and Biochemistry, University of Windsor, Windsor, ON N9B 3P4, Canada; ruedam@uwindsor.ca; 3 King Hussein School of Computing Science, Princess Sumaya University for Technology, Al-Jubaiha, Amman P.O. Box 1438, Jordan

**Keywords:** breast cancer, prognosis, survival, residual neural network, high-dimensional embedding, classification, bioinformatics, multi-omics

## Abstract

**Simple Summary:**

A deep learning model based on multi-omics data to classify Nottingham prognostic Index score levels. The model represents each omic dataset using 2-dimensional map before integrating all omics maps into the prediction model. The literature confirms the relationship between the extracted omics features with the progression and survival of breast cancer.

**Abstract:**

The Nottingham Prognostics Index (NPI) is a prognostics measure that predicts operable primary breast cancer survival. The NPI value is calculated based on the size of the tumor, the number of lymph nodes, and the tumor grade. Next-generation sequencing advancements have led to measuring different biological indicators called multi-omics data. The availability of multi-omics data triggered the challenge of integrating and analyzing these various biological measures to understand the progression of the diseases. High-dimensional embedding techniques are incorporated to present the features in the lower dimension, i.e., in a 2-dimensional map. The dataset consists of three -omics: gene expression, copy number alteration (CNA), and mRNA from 1885 female patients. The model creates a gene similarity network (GSN) map for each omic using t-distributed stochastic neighbor embedding (*t*-SNE) before being merged into the residual neural network (ResNet) classification model. The aim of this work was to (i) extract multi-omics biomarkers that are associated with the prognosis and prediction of breast cancer survival; and (ii) build a prediction model for multi-class breast cancer NPI classes. We evaluated this model and compared it to different high-dimensional embedding techniques and neural network combinations. The proposed model outperformed the other methods with an accuracy of 98.48%, and the area under the curve (AUC) equals 0.9999. The findings in the literature confirm associations between some of the extracted omics and breast cancer prognosis and survival including *CDCA5*, *IL17RB*, *MUC2*, *NOD2* and *NXPH4* from the gene expression dataset; *MED30*, *RAD21*, *EIF3H* and *EIF3E* from the CNA dataset; and *CENPA*, *MACF1*, *UGT2B7* and *SEMA3B* from the mRNA dataset.

## 1. Introduction

Breast cancer (BC) is one of the most common cancers and leading cause of cancer death in women worldwide [1], accounting for 30% of female cancers [2]. BC is a heterogeneous disease that consists of several subtypes which have been identified at the clinical, molecular, genomic, and histological levels. Early detection and intervention are proven to be effective in increasing survival rates and improving prognosis [3]. The Nottingham Prognosis Index (NPI) is an index to determine prognosis following surgery for BC [4]. Various studies have validated the prognostic discrimination [5,6,7] and NPI is widely used in clinical practice. It is a well-established prognostic classification scheme for patients with breast cancer [8]. NPI combines the nodal status, tumor size and histological grade in a simple formula:

(1) NPI=[0.2×S]+N+G

where: 

*S* is the size of the index lesion in centimeters;

*N* is the node status—0 nodes = 1, 1–3 nodes = 2, >3 nodes = 3;

*G* is the grade of the tumor—grade I = 1, grade II = 2, and grade III = 3 [9]. 

Understanding the prognosis of BC is vital for selecting the right treatment [10]. Patients with a very poor prognosis may be considered for aggressive treatment, or those with excellent prognosis may receive light treatment. These practices lead to health side-effects and financial costs [10,11]. In clinical practice, NPI is an important index that influences the decision of whether or not to undergo adjuvant chemotherapy following surgery. Recent studies have indicated that NPI still outperforms other prognostics models [10,12]. Phung et al. studied 58 prognosis models which predict mortality, recurrence, or both. Most of the models perform well in most cohorts but are less accurate on the independent populations, except NPI, which retains its prediction ability in most independent populations [10]. Sejben et al. studied the survival of 136 triple-negative breast cancer (TNBC). TNBC is generally known as a poor prognosis BC subtype. The study compared three prognostics models that are NPI, PREDICT [13] and PrognosTILs [14]. None of the three models are inferior to the others; however, NPI outperforms the others in the area under the curve (AUC) prediction measurement [12].

Machine learning models have also been used in breast cancer prognosis prediction [15,16]. Boeri et al. investigated the standard machine learning classifiers to predict three outcomes that are loco-regional cancer recurrence, systematic cancer recurrence, and death from the disease within 32 months. The results show that the artificial neural network (ANN) and support vector machine (SVM) outperformed some other standard classifiers with an accuracy higher than 95% for the three classes [15]. Ferroni et al. applied a machine learning model based on demographic, clinical, and biochemical data to predict breast cancer survival. The model incorporated multiple kernel learning (MKL) based on support vector machines (SVMs) combined with random optimization (RO) models to extract the discriminative features and build the classification model. The results show that NPI was one of the clinical features that strongly influenced survival [16].

In an earlier work, we proposed a ResNet model based on the *t*-SNE embedding method to classify two classes of patients; with NPI<3.4 versus NPI≥3.4 where 3.4 is the cut-off between the high survival rate and low survival rate. The model was applied on two omics that are gene expression and CNA [17]. In this work, we extending the prediction criteria to include all NPI classes that are seen in Table 1. The idea is to extract potential omics biomarkers for all levels of NPI values.

## 2. Materials and Methods

### Materials

For the selected samples, we extracted three omics from the METABRIC dataset which were gene expression, mRNA, and copy number alteration (CNA) [18]. The numbers of features for all omics are listed in Table 2.

The clinical characteristics of the study population such as hormone receptor expression, HER2 status, adjuvant treatments, and tumor size and laterality among other features can be found on this portal (https://www.cbioportal.org/study/clinicalData?id$=$brca_metabric accessed on 2 February 2022).

## 3. Methods

The methods section starts with pre-processing the dataset, applying a hybrid feature selection approach, data embedding, and the prediction model.

### 3.1. Pre-Processing

Genome-wide data have the characteristics of high-dimensionality. In addition, it also suffers from data incompleteness due to sequencing technology or loss of biological information. To keep the reliability of the data, we dropped the patients with missing values into three omics datasets. As a result, the dataset ended up with 1885 samples.

### 3.2. Feature Selection

A hybrid feature selection approach was applied to select each omic dataset’s best possible subset of features. It consists of ranker and wrapper feature selection methods. The ranker chi-square was applied to the three omics datasets to choose 1000 top genes, followed by the wrapper that works as the following:

***Chi-Square*** Pearson’s chi-squared test, called chi-square [19], is a statistical ranker-based feature selection method that selects top *N* features that are sensitive to the label. Equations (2)–(4) show how chi-square works, by which we decided to select the top 1000 features from 16,041 features in the gene expression dataset. For a specific gene, we first sum up the expression level according to the label as the observed values. It then counts the number of samples belonging to each class, divided by the total number of samples, which is the class probability. The class probabilities are multiplied by the sum of the expression level of that gene, and we call the result the expected value.

Finally, the square of the difference between the observed value and the expected value is divided by the expected value. The sum of the obtained results is the chi value of the individual gene. In our experiment, we picked up the top 1000 genes:

(2) $$O_{jc}=\sum_{i=i}^{n}x_{ijc}$$

(3) $$E_{jc}=\frac{n_{c}}{N}\times \sum_{i=1}^{N}X_{ij}$$

(4) $$\chi 2\left(X_{j},Y\right)=\sum_{c=1}^{k}\frac{{\left(O_{jc}-E_{jc}\right)}^{2}}{E_{jc}}$$

where: 

$n_{c}$ is the number of samples belonging to class *c*;

*i* is the *i*th sample, i∈{1,2,…,N};

*j* is the *j*th feature, j∈{1,2,…,m};

*c* is the *c*th class, c∈{1,2,…,k};

*N* is the total number of samples. 

***mRMR*** is a wrapper feature selection algorithm that finds a compact subset of superior features with the characteristics of maximal relevance to the class, that is max *D*(*S*,*c*) and minimal redundancy in the feature subset, that is, min *R*(*S*), according to the maximal statistical dependency criterion based on mutual information, described in Equations (5) and (6):

(5) $$maxD\left(S,c\right),D=\frac{1}{\left|S\right|}\sum_{x_{i}\in S}I\left(x_{i},c\right)$$

(6) $$minR\left(S\right),R=\frac{1}{{\left|S\right|}^{2}}\sum_{x_{i},x_{j}\in S}I\left(x_{i},x_{j}\right)$$

where: 

*S* is the subset of superior features;

*c* is the label class;

$x_{i}$, $x_{i}$ are the sample vectors of the *i*th and *j*th features, respectively, with the shape of 1 × *N* (*N* is the number of samples). 

In this work, mRMR incorporates the random forest classifier [20] with forward search feature selection to obtain the best possible subset of features that represent each omic dataset.

### 3.3. *t*-SNE

*t*-SNE is a non-linear dimensionality reduction technique used to embed high-dimensional data points into a lower-dimensional space to seek a pattern that represents the original feature map [21]. The feature map reflects the underlying relationship among the marker genes in terms of biological pathway or functionality in three datasets, which is called the gene similarity network (GSN) map. The Euclidean distance in the high-dimensional space decides the pairwise similarities among samples in the lower dimensional space. *t*-SNE applies a *t*-student distribution to avoid a crowding problem as shown in Equations (7) and (8):

(7) $$p_{ij}=\frac{p_{i|j}+p_{j|i}}{2n},\;\;p_{ij}\neq p_{ji}\;\;for\;\;\forall \;\;i,\;j$$

where: 

$p_{ij}$ is the joint probability in the high dimensional space;

$p_{i|j}$ and $p_{j|i}$ are the conditional probability in the high dimensional space. 

In the high dimensional space:

(8) $$q_{ij}=\frac{{\left(1+{\left|\right|y_{i}-yj\left|\right|}^{2}\right)}^{-1}}{\sum_{k\neq l}{\left(1+{\left|\right|y_{k}-yl\left|\right|}^{2}\right)}^{-1}},\;\;q_{ij}\neq q_{ji}\;\;for\;\;\forall \;\;i,\;j$$

where: 

$q_{ij}$ is the joint probability in the low-dimensional space:

(9) $$\frac{\delta C}{\delta y_{i}}=4\sum_{j}\left(p_{ij}-q_{ij}\right){\left(1+{\left|\right|y_{k}-yl\left|\right|}^{2}\right)}^{-1}\left(y_{i}-yj\right)$$

where: 

$\frac{\delta C}{\delta y_{i}}$ is the gradient descent. 

### 3.4. ResNet

In a residual neural network [22], the aim is to find a function $f^{*}$ in the class of function *F* to best represent the relationship between dataset *X* and its label *y*, which means that the distance between *X* and *y* is minimal, as shown in Equation (12). In a residual neural network, He et al. suggested an optimal function in the output layer. It can be expressed as the sum of identity mapping and the residual function as shown in Equation (10) [22]:

(10) H(x)=F(x)+x

where: 

H(x) is the optimal function;

F(x) is the residual function;

*x* is the identity mapping. 

**Figure 1:**
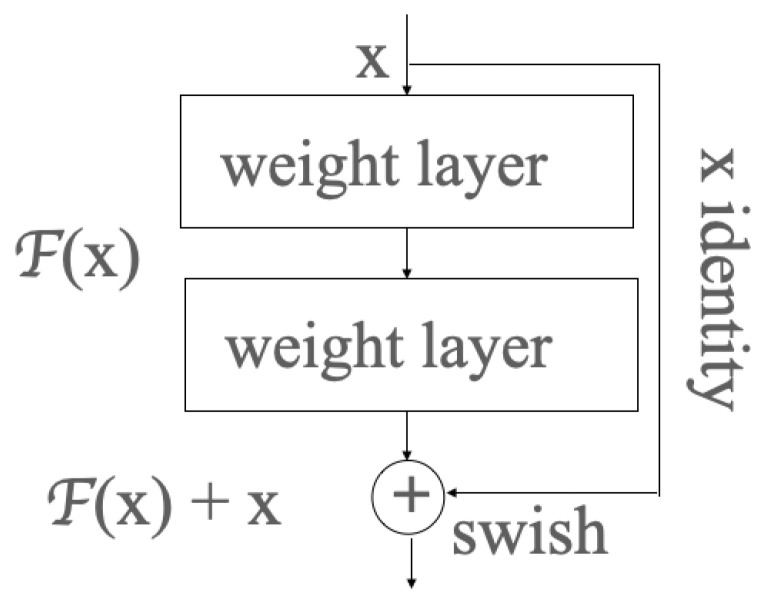
One block in the residual neural network.

In ResNet, the residual learning is applied to every few stacked layers, which is called a block shown in Figure 1. In the output layer of each block, there is an identity mapping by shortcuts, and the output *y* can be expressed by the product dot of the weight vector and input *x*, before being added to the identity mapping *x*, as shown in Formula (11):

(11) Y=F(x)+x

where: 

*X* is the input vector;

*Y* is the output vector;

$F(X,\left\{W_{i}\right\})$ represents the residual mapping to be learned:

(12) $$f_{F}^{*}\;\overset{\mathrm{def}}{=}\;argimL\left(X,y,F\right)\;Subject\;to\;f\in F$$

where: 

*X* is the input data;

*y* is the label or class;

$f^{*}$ is the function, in which X can find its minimal distance to *y*;

*F* is the class of functions comprising many functions to represent the pattern between input data and labels.

### 3.5. SOM

SOM is a simple neuron network algorithm that allows us to investigate the intrinsic relationships among samples of a dataset. As such, the data points with the smallest weights to the winning neuron will be grouped in the same cluster, forming the GSN as a result, then colored by the transformed sequencing values of the three omics datasets in a merged way or a concatenated way.

**Figure 2:**
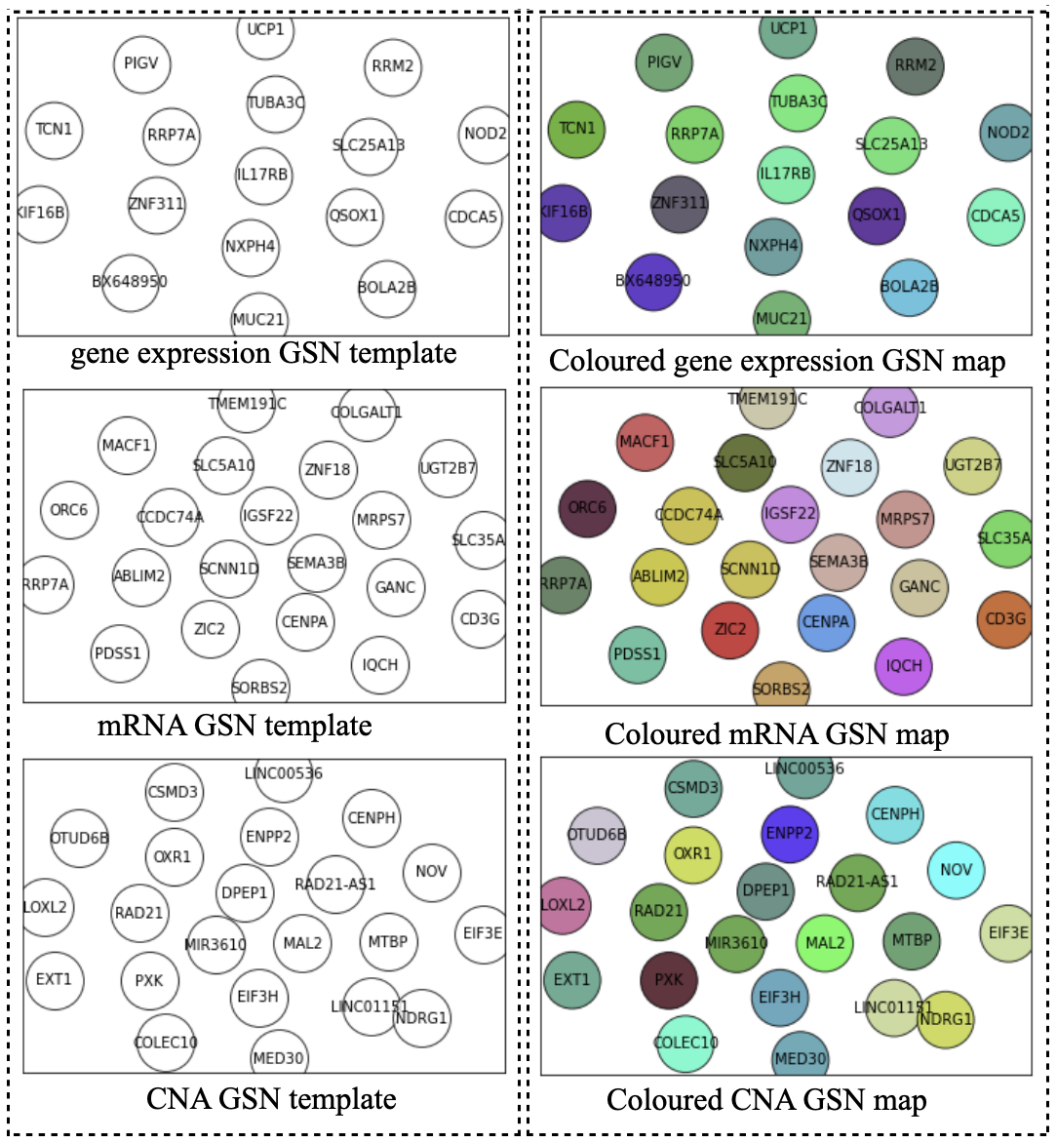
Templates of three omics datasets embedded by *t*-SNE (concatenated).

**Figure 3:**
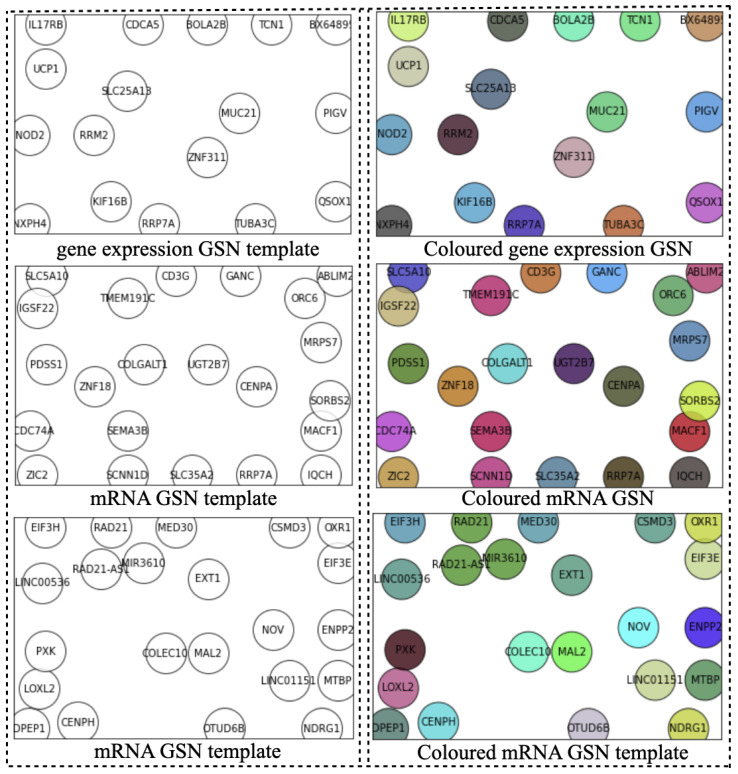
Templates of three omics datasets embedded by SOM (concatenated).

**Figure 4:**
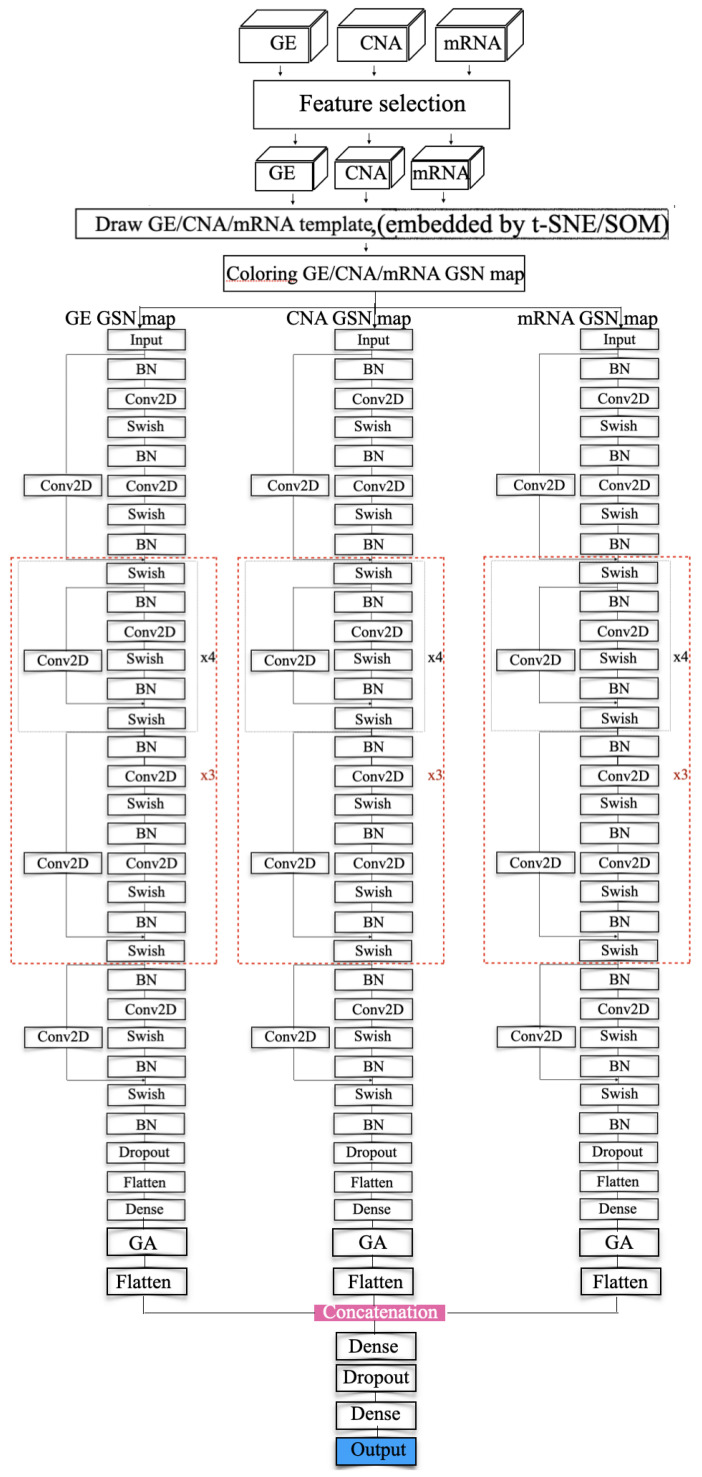
Resnet modeling (concatenated).

Ideally, the GSN reveals the closeness of the most informative genes in terms of biological pathways or functionality. Therefore, the “colored template” represents each NPI class based on the relationship among their marker genes. Figure 2 and Figure 3 show the *t*-SNE–GSN and SOM–GSN template creating and coloring in a concatenated way model that is seen in Figure 4. In contrast, the marker genes in the *t*-SNE–GSN map have an even distribution, leading to a capacity to hold more marker genes inside than the SOM–GSN map.

## 4. Experiments and Results

To keep the highest possible meaningful information in the three omics datasets, the hybrid feature approach selected 1000 genes using chi-square for each omic dataset. Then, the wrapper method selected 17 genes from the gene expression dataset, 22 genes from the mRNA dataset, and 22 target genes from the CNA dataset separately, as shown in Table 3.

**Figure 5:**
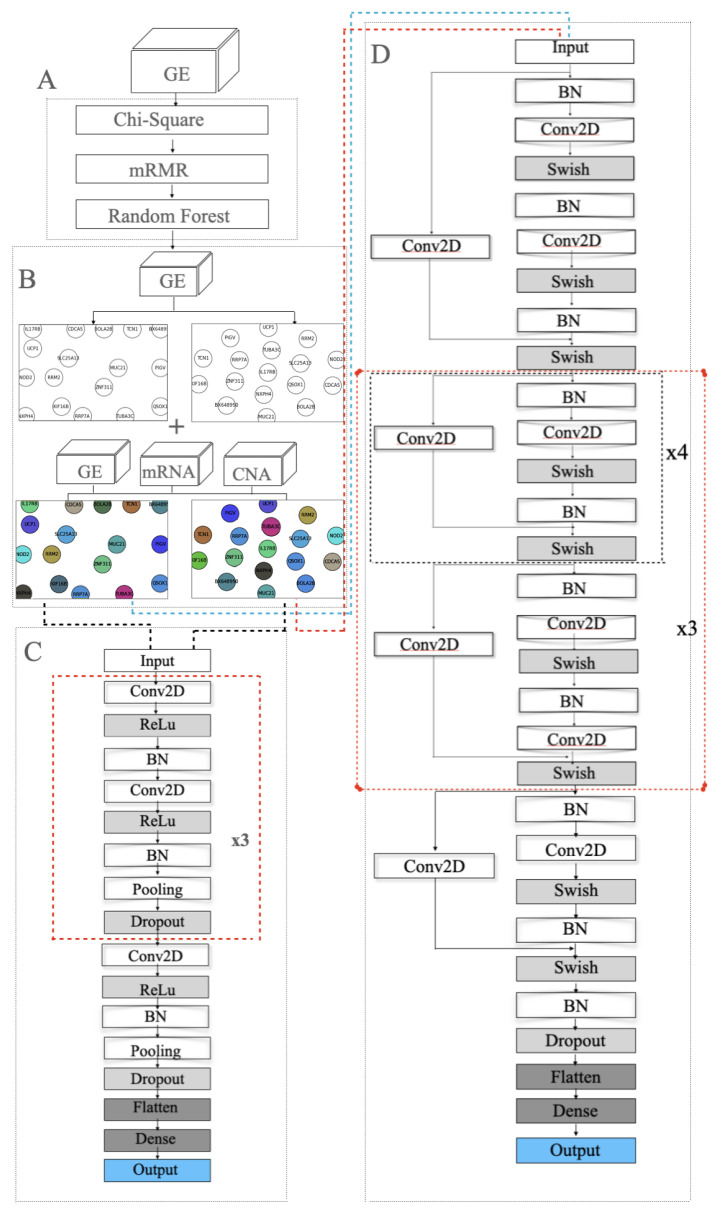
Modelling in the merged approach: (**A**) feature selection using chi-square, mRMR and random forest; (**B**) SOM and *t*-SNE embedding to draw two 2D templates of the 22 selected genes. The left template was made by SOM and the right template was drawn by *t*-SNE. The templates are colored by combining the three omic datasets; (**C**) the 33-layer VGG model; (**D**) the 112-layer ResNet model.

In the second step, we drew the GSN maps using *t*-SNE and SOM embedding methods separately for each dataset in a concatenated way, or only using the gene expression dataset in a merged way as seen in Figure 2, Figure 3 and Figure 5B. The left template is created by SOM, and the right template is drawn by *t*-SNE. Then, the templates are colored by combining the three omic datasets in Figure 5.

In the third step, we colored the GSN maps for each sample in the three omic datasets separately. In a concatenated way, the color is the helix values of the normalized genes by logarithmic and z-score normalization methods. In the merged way, the color is the RGB values of the normalized sequencing data, in which R represents the gene expression dataset, G represents the CNA dataset, while B represents the mRNA dataset. Therefore, the colored GSN maps reflect the relationships among the genes in each sample, the characteristics of the position, and the pixel.

Before feeding the ResNet model with the GSN maps as the inputs, the number of colored GSN maps was boosted by applying data augmentation, intending to solve the imbalanced problem and increase the recognition rate. Elastic argumentation was utilized and alpha was set to be 15–18. The new dataset seems fine; the characteristics are in a reasonable range compared with the original images.

In the training step, several models were investigated including VGG and ResNet. Figure 5C,D show the VGG model and modified ResNet model that were applied to train the GSN maps that were embedded by SOM and *t*-SNE in the merged approach. Figure 4 depicts the proposed model that concatenates three ResNet models to train the three collections of GSN maps, separately extracting the features of position and pixel. Then, the features are combined and build the final classification model.

The ResNet model is an extension of the VGG model, but it removes the dropout layer, pooling layer and adds a one-by-one ConvNet layer to the end of the ResNet block. The model ended up having a ResNet model that consists of 112 layers. In the beginning, batch normalization normalizes the image pixels and feeds the normalized inputs to a ConvNet layer with “same” padding layer to protect the marginal features and the “Swish” activation function to smoothly transform the inputs and repeat. Then the mapping layer, which is another difference with VGG, is added to the end to calculate the residual and optimize the weights. After that, a four-layer ResNet block with a 1 × 1 ConvNet mapping layer is repeated four times and followed by a five-layer ResNet block. The former blocks are repeated three times. Then, a four-layer of the ResNet block is added. At the end of the feature-extraction process, the result is normalized again.

In the classification process, we adopted a flattened layer and a dense layer as VGG does. In the end, the softmax probability function is used for the prediction.

The model has two kinds of ResNet blocks with four and five layers, respectively. Each ConvNet layer has a 7 × 7 kernel, and the stride size is 1. The ConvNet layer contains a 1 × 1 kernel in the mapping layer, and the stride size is 2 for downsampling. At the end of the feature extraction, the VGG model incorporates a flatten layer and fully connected layer with the softmax probability function to output a 5D array with values ranging from 0 and 1.

The ResNet model incorporates the average pooling layer instead of the fully connected layer. The loss functions in the two different models are categorical entropy loss functions. The model uses "accuracy" and "AUC" as performance metrics. It extensively extracts the features and classifies the inputs using softmax for probability calculation. Equation (13) shows the softmax probability function:

(13) $$P\left(y=j|X\right)=\frac{e^{X^{T}w_{j}}}{\Sigma_{k=1}^{K}e^{X^{T}w_{j}}}$$

where: 

*X* is the sample vector;

$w_{j}$ is the weight vector of *j*th feature;

*K* is the number of features—which here is 22.

Table 4 and Table 5 show the performance of our proposed model and other similar models for comparison. Among eight different models, the models in the concatenated way have higher accuracy and AUC, but lower loss in the training process. Conversely, predicting performance in the merged way outperforms that in the concatenated approach since the models have higher validation accuracy.

## 5. Conclusions

The Nottingham Prognosis Index (NPI) is a prognosis measure employing following surgery for BC. In this work, multi-omics features that are associated with NPI classes were extracted using a hybrid feature selection method. The selected features were represented in 2-dimensional GSN maps using the *t*-SNE method. The maps from different omics were fed into a deep residual neural network for classification.

The main limitation of this work was not considering more clinical features in the analysis including race, age, and response to different therapies. From the computational side, the model can be more flexible to add more sources of data (omics). A future work can be to try the Mahalanobis instead of Euclidean distance in the *t*-SNE GSN mapping, since it has recently been used in prognostics and survival predictions with interesting results [23,24].

## 6. Biological Insight

Genes extracted from the gene expression omic dataset that play key roles in the development of breast cancer are *CDCA5*, *IL17RB*, *MUC2*, *NOD2*, and *NXPH4*. *CDCA5* is a cell division cycle-associated (*CDCA*) gene family member. Specifically, it controls sister-chromatid cohesion and separation during cell division. Phan et al. screened the mRNA expression of the *CDCA* genes and found that the expression of *CDCA5*, along with *CDCA3* and *CDCA8*, was significantly high compared to the control sample. The high expression of these genes in tumors dramatically reduced patient survival, which has the potential for being a target for breast cancer therapies [25]. According to another study by Zhang et al., *CDCA5* also has the potential to become a prognostic strategy because it has been found to be significantly upregulated in gastric cancer (GC) tissues. When *CDCA5* was inhibited in gastric cancer cells, it suppressed their proliferation, demonstrating how *CDCA5* promotes GC tumor progression [26].

*IL17RB* is a member of the *IL17* gene family. *IL17* genes encode pro-inflammatory cytokines produced from inflammatory diseases such as breast cancer. One of the *IL17* genes is *IL17B*, which binds to its receptor, *IL17RB*. The *IL17RB* signaling pathway plays a key role in its progression, so targeting this pathway is a potential therapeutic pathway for breast cancer inhibition [27]. Alinejad et al. demonstrated that the *IL17RB* signaling pathway triggers an increase in cell growth through the activation of NF-KB and Bcl-2 [27].

*MUC2* is a member of the Mucins family, which are glycoproteins expressed and secreted as mucus by epithelial cells and their malignant counterparts [28]. *MUC2* is a protein that is highly expressed in mucin secreted breast cancers. *MUC2* plays an important role in mediating the proliferation, apoptosis and metastasis of breast cancer [29].

*NOD2* is an innate immune receptor that is associated with the onset and progression of triple-negative breast cancer (TNBC). The overexpression of *NOD2* is shown to reduce cell proliferation. The pathways that link *NOD1* and *NOD2* signaling to tumorigenesis have been studied. Velloso et al. found that the up- and downregulation of inflammatory proteins can promote protein degradation systems and modulate cell cycle and cell adhesion proteins [30].

*NXPH4* is a mitochondria-related core gene involved in the progression of breast cancer. Yan et al. found that *NXPH4* is upregulated in breast cancer, which can be used as a potential biomarker for breast cancer prognosis and diagnosis [31].

One of the genes found in the CNA omic dataset is *MED30*, a core subunit in a group of mediator complex subunits that are mutated in various malignancies in glioblastoma (GBM), a malignant form of glial cell cancer. *MED30* was found to be overexpressed in GBM tissues and cell lines, and was found to be induced by conditions present in tumor environments, such as hypoxia. *MED30* contains hypoxia response elements (HREs) and a p53 binding site in its promoter region, showing that *MED30* promotes cell proliferation while reducing migration capabilities in GBM cell lines. Its involvement in GBM pathogenesis has also revealed that *MED30* is sensitive to the chemo drug temozolomide, suggesting its diagnostic and therapeutic potential [32]. *MED30* has also been reported to be overexpressed in various breast cancer cell lines, along with *MED1* and *MED24* [33].

*RAD21* is a gene that is believed to function in sister chromatid alignment and double-stranded break repair. Expression studies have revealed a 2-fold increased expression of *RAD21* in human breast cancer cell lines compared to normal breast tissue. Atienza et al. conducted experiments with RNA interference technology and found that the proliferation of cells with *RAD21*-specific siRNA was reduced. This suppression of the *RAD21* gene also enhanced the cytotoxicity of etoposide and bleomycin (two DNA-damaging chemotherapeutic agents), which revealed that by the induction of DNA damage, *RAD21* can be a novel target for developing cancer therapeutics [34].

*EIF3H* is a gene that is involved in the pathway for the metabolism of proteins [35] and it is involved in the translation inhibition factor activity. Mahmood et al. performed siRNA screening in three breast cancer cell lines for candidate driver genes that are overexpressed in breast tumors. This study identified eight driver genes that were amplified and critical for breast tumor cell proliferation, including the *EIF3H* gene and some other well-described oncogenic drivers *ERBB2*, *GRB7*, *RAD21 CHRAC1*, and *TANC2*. Therefore, strategies for inhibiting the expression of these genes are of potential value for the treatment of breast cancers [36].

Another potential gene biomarker in the CNA dataset is *EIF3E*, a translation factor associated with breast cancer occurrence which is related to *EIF3H*. It has been previously shown that *EIF3E* deficiency leads to an impaired DNA damage response along with a marked decrease in DNA repair via homologous recombination. Mahmood et al. found that *EIF3E*-depleted cells synthesized lower amounts of PARP1 protein, and the mTORC1 signaling pathway is more activated. These PARP1 and mTORC1 dysfunctions are linked to the induction of cellular senescence, which provides mechanistic insights into how *EIF3E* protects against breast cancer. Findings suggest that these cancers may benefit from mTORC1 inhibitor drugs [37].

Another gene found in the CNA dataset is *MAL2*, a gene identified to determine the turnover of the antigen-loaded MHC–I complex and reduces antigen presentation on a tumor cell, which promotes immune evasion. Fang et al. showed how *MAL2* accomplishes this by promoting the endocytosis of tumor antigens by directly interacting with the MHC–I complex and endosome-associated RAB proteins. It has been shown that the suppression of *MAL2* has enhanced the cytotoxicity of tumor-infiltrating CD8+ T cells and suppressed breast tumor growth, which suggests that this gene can be a potential biomarker for breast cancer therapy [38]. Genes extracted from the mRNA dataset, which show great significance in the development of breast cancer, are *CENPA*, *MACF1*, *UGT2B7* and *SEMA3B*. Centromere protein-A, also known as *CENPA*, is a histone-like gene that is important for regulating chromosome segregation that occurs during cell division. It is a commercially available gene for prognostic breast cancer assays. Rajput et al. found that by measuring the immunohistochemical (IHC) expression of *CENPA* in tissues, breast cancer tissue had higher levels of IHC than normal breast tissue. They found that a higher IHC expression was correlated with a shorter disease-free survival (DFS), making *CENPA* a potential prognostic biomarker [39].

Microtubule actin cross-linking factor 1, also known as *MACF1*, is a gene that plays an essential role in various cellular processes such as proliferation and embryo development. It is also known to be a gene responsible for the development of breast cancer. *MACF1* also plays a role in cytoskeleton organization in cells, which contributes to tumor progression, and the abnormal expression of *MACF1* initiates proliferation, metastasis, and migration in breast cancer [40].

Uridine glucuronosyltransferase 2B7, also known as *UGT2B7*, is a gene that belongs to the *UGT* gene family, and it plays an essential role in the elimination of toxic xenobiotics and endogenous compounds. It is found in the microsome membrane [41]. Li et al. studied the occurrence of cytotoxicity in breast cancer patients undergoing chemotherapy along with the expression of the *UGT2B7* gene. Their results showed that breast cancer patients with the *UGT2B7* gene had a lower occurrence of cardiotoxicity [42]. It has also been found by Mou et al. that a gene in the *UGT2B7* gene called *RS745335* was associated with breast cancer patients receiving chemotherapy, therefore making *RS745335* and *UGT2B7* potential biomarkers for breast cancer [43].

Semaphorin 3B, also known as *SEMA3B*, is a gene that belongs to the semaphorin family and is important in the process of growth cone guidance during neuronal development [44]. Castro-Rivera et al. studied how *SEMA3B* acts as a tumor suppressor by inducing apoptosis. They found that another growth factor called *VEGF165* binds to neuropilin, which is also a receptor for *SEMA3B*. It decreases the proapoptotic effect that *SEMA3B* had since it will competitively bind to those receptors. Therefore, it was concluded that *VEGF165* was produced by tumor cells and was used as a surviving factor, and *SEMA3B* has tumor-suppressing effects by blocking this VEGF pathway [45]. In a related study by Castro-Rivera et al., it was found that *SEMA3B* induces apoptosis by inactivating the Akt signaling pathway through the Np-1 receptor [46].

**Figure 6:**
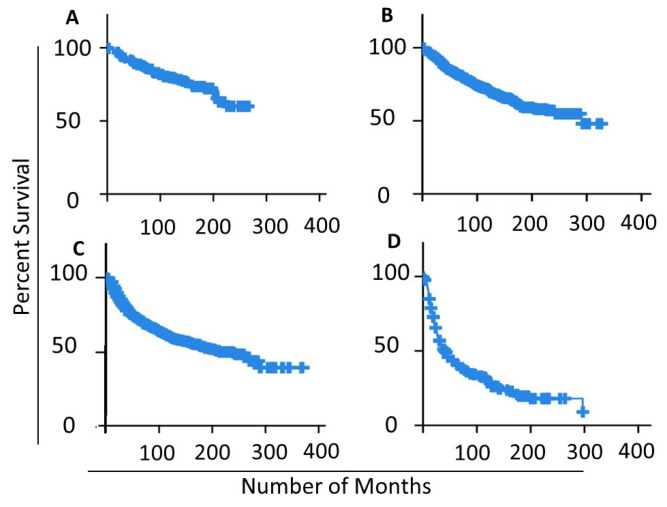
Kaplan–Meier curve for different NPI classes in the studied samples: (**A**) NPI Level I samples; (**B**) NPI Level II samples; (**C**) NPI Level III samples; (**D**) NPI Level IV samples.

To validate the used NPI classes as significant prognostics and survival patterns, we applied the Kaplan–Meier curve to estimate the relapse survival rate for each NPI class in the dataset as seen in Figure 6. The average relapse-free survival for NPI levels I, II, III and IV are 120, 125, 109 and 63 months accordingly. Figure 6D depicts the drastic drop in survival periods for NPI level IV samples, which explains the drop in the overall average 63 months down from 109 in level III. Relapse-free status includes those cases which in which there was recurrent loco-regional relapse, distant relapse, or death-specific death. The Figure confirms the different patterns of survivability for the NPI classes.

The advancement in NGS and artificial intelligence yield integrate multi-omics measurements in the prediction model. The literature analysis confirms the association between the extracted omics and the survival and prognosis of breast cancer. However, more wet-lab and data analysis of the extracted omics can verify the role of this subset of omics in the progression of the disease.

## Figures and Tables

**Table 1:** NPI levels and their 5-year survival rate.

Level	Score	Survival-Rate
I	from ⩾2.0 to ⪕2.4	93%
II	from >2.4 to ⪕3.4	85%
III	from >3.4 to ⪕5.4	70%
IV	>5.4	50%

**Table 2:** Datasets after sample and gene mapping.

Dataset Name	Number of Genes	Number of Samples
Gene expression	16,567	1885
mRNA	16,567	1885
CNA	16,401	1885

**Table 3:** Target genes in three datasets.

17 Target Genes in Gene Expression Dataset.							
**NO**	**Gene**	**NO**	**Gene**	**NO**	**Gene**	**NO**	**Gene**
01	CDCA5	06	TUBA3C	11	PIGV	16	RRM2
02	IL17RB	07	BX648950	12	TCN1	17	QSOX1
03	MUC2	08	NXPH4	13	BOLA2B		
04	ZNF311	09	UCP1	14	KIF16B		
05	NOD2	10	SLC25A13	15	RRP7A		
**22 Target Genes in mRNA Dataset.**							
**NO**	**Gene**	**NO**	**Gene**	**NO**	**Gene**	**NO**	**Gene**
01	CENPA	07	IGSF22	13	ZNF18	19	ABLIM2
02	SLC5A10	08	RRP7A	14	ORC6	20	SORBS2
03	CD3G	09	SLC35A2	15	TMEM191C	21	PDSS1
04	MACF1	10	CCDC74A	16	SEMA3B	22	GANC
05	SCNN1D	11	MRPS7	17	ZIC2		
06	UGT2B7	12	COLGALT1	18	IQCH		
**22 Target Genes in CNA Dataset.**							
**NO**	**Gene**	**NO**	**Gene**	**NO**	**Gene**	**NO**	**Gene**
01	RAD21-AS1	07	EIF3H	13	NOV	19	ENPP2
02	PXK	08	LINC01151	14	NDRG1	20	OTUD6B
03	MED30	09	LINC00536	15	CSMD3	21	MTBP
04	MIR3610	10	EXT1	16	MAL2	22	CENPH
05	LOXL2	11	EIF3E	17	DPEP1		
06	RAD21	12	COLEC10	18	OXR1		

**Table 4:** Performance comparison among classifiers and embedding methods (merged).

Method	Classifier	Acc	Val_Acc	Loss	Val_Loss	AUC	Val_AU
SOM	VGG-33	0.8972	0.87858	0.2539	0.21189	0.9913	0.97
SOM	ResNet-112	0.9702	0.8653	0.1062	0.2647	0.9985	0.9649
*t*-SNE	VGG-33	0.9384	0.8265	0.31	0.2210	0.9845	0.8979
*t*-SNE	ResNet-112	0.9609	0.8371	0.1471	0.2797	0.9958	0.9179

**Table 5:** Performance comparison among classifiers and embedding methods (concatenated).

Method	Classifier	Acc	Val_Acc	Loss	Val_Loss	AUC	Val_AU
SOM	VGG-33	0.9375	0.75	0.2044	1.043	0.9956	0.8662
SOM	ResNet-112	0.9375	0.75	0.2044	1.043	0.9956	0.8662
*t*-SNE	VGG-33	0.9773	0.8	0.0546	0.7122	0.9999	0.9625
*t*-SNE	ResNet-112	0.9848	0.8	0.0972	0.5909	0.9991	0.9500

## Data Availability

The origincal dataset can be found at the following link: (https://www.cbioportal.org/study/clinicalData?id$=$brca_metabric, accessed on 2 February 2022).

## Abbreviations

The following abbreviations are used in this manuscript:

NPI	Nottingham Prognostic Index
CNA	Copy Number Alteration
*t*-SNE	t-Distributed Stochastic Neighbor Embedding
GSN	Gene Similarity Network
